# Predictors of Psychosocial Functioning Among Long-stay Schizophrenia Patients in a Malaysian Mental Institution

**DOI:** 10.21315/mjms2024.31.6.14

**Published:** 2024-12-31

**Authors:** Pey Fang Teo, Eugene Boon Yau Koh, Seng Choi Chong

**Affiliations:** 1 Department of Psychiatry, Hospital Pakar Sultanah Fatimah, Johor, Malaysia; 2 Department of Psychiatry, Faculty of Medicine and Health Sciences, Universiti Putra Malaysia, Selangor, Malaysia

**Keywords:** long-stay, schizophrenia, mental institution, psychosocial functioning, cognition, negative symptom

## Abstract

**Background:**

A considerable number of schizophrenia patients still require long-term hospital care despite psychiatric deinstitutionalisation, especially in developing nations. Prolonged hospitalisation is associated with greater impairment in psychosocial functioning. This study aimed to determine the level of psychosocial functioning and its predictors among long-stay schizophrenia patients in a Malaysian mental institution.

**Methods:**

This cross-sectional study included 138 patients selected through universal sampling. Data on socio-demographics, illness characteristics such as psychopathology and illness severity [measured using the Brief Psychiatric Rating Scale (BPRS)], and cognitive function [assessed using the Montreal Cognitive Assessment (MoCA)] were collected. The Personal and Social Performance (PSP) scale was used to evaluate psychosocial functioning. Pearson correlation coefficients and multiple linear regression analyses were applied to identify the correlates and predictors of psychosocial functioning.

**Results:**

This study found that 47.8% and 16.7% of the patients had moderate and severe cognitive impairments, respectively. The mean PSP score was 69.68 (standard deviation (SD) = 15.48). Female gender, previous unemployment and more severe cognitive impairments were significantly associated with poorer psychosocial functioning. Meanwhile, negative symptoms and age of onset were negatively correlated with psychosocial functioning. By contrast, the duration of illness was positively correlated with psychosocial functioning. The regression model indicated that being female (β = −7.32, *p* < 0.001), previously unemployed (β = −3.67, *p* < 0.047), having negative symptoms (β = −4.18, *p* < 0.001), experiencing a longer illness duration (β = −0.60, *p* = 0.004), and the presence of severe cognitive impairment (β = −9.80, *p* < 0.001) significantly predicted poorer psychosocial functioning.

**Conclusion:**

Long-stay schizophrenia patients experience substantial difficulties in psychosocial functioning. Factors such as gender, last employment status, negative symptoms, illness duration, and cognitive function affect psychosocial functioning.

## Introduction

Schizophrenia is a chronic and debilitating mental disorder that not only affects individuals’ mental health but also significantly impacts their psychosocial functioning, resulting in a substantial annual economic burden estimated between USD94 million and USD102 billion globally (1). In Malaysia, psychiatric hospitals incur a total direct medical cost of about USD26 million, with 83% of this expenditure allocated to long-stay hospital services (USD12 million) (2). Despite psychiatric deinstitutionalisation, a considerable number of schizophrenia patients continue to require long-term hospitalisation, particularly in developing nations. Long-stay schizophrenia patients represent a distinct population within mental healthcare settings, often characterised by severe functional impairment and social isolation. They also face numerous challenges that hinder their psychosocial functioning and successful reintegration into the community (3).

The definition of “long-stay” varies across different studies (4, 5). However, it generally refers to patients who require extended hospitalisation, often exceeding six months (6). Various studies worldwide, including those in the UK, Australia, Korea, and Malaysia, have established the prevalence of long-stay psychiatric patients, with schizophrenia being the most common diagnosis among this group (6–9). According to the annual report from Hospital Bahagia Ulu Kinta (HBUK)—the first and largest mental institution in Malaysia—the proportion of schizophrenia patients among all psychiatric inpatients was 55.4% in 2021 (10).

Prolonged hospitalisation is associated with greater impairment in psychosocial functioning, encompassing an individual’s ability to engage in daily activities, maintain relationships and meet societal demands (11–13). Global functional impairment is a well-documented key issue among schizophrenia patients, leading to challenges in social, occupational, and personal domains (3, 13–16). Successful treatment of schizophrenia is defined not only by symptom remission but also by improvements in psychosocial functioning, which has become the ultimate goal of treatment. Given that a significant extent of preventive or therapeutic interventions relies on targeting the associated factors, numerous studies have been conducted to examine this aspect. However, many existing studies primarily focus on schizophrenia patients in general or during the acute stage, with limited attention given to long-stay patients (16–19).

Current evidence indicates that being younger, unemployed, experiencing a long duration of illness or an extended length of the current episode, and suffering from severe negative symptoms are predictors of poor psychosocial functioning in schizophrenia (13). Cognition also plays a crucial role in daily functioning and has been identified as a significant predictor of functional outcomes in schizophrenia (20–30). Notably, older patients with a long duration of illness and protracted institutionalisation have shown a more pronounced progressive cognitive decline (31).

Although substantial information is available on the determinants of functional outcomes in schizophrenia, there exists a notable gap in the literature regarding the predictors of psychosocial functioning among Asian patients receiving long-term care in mental hospitals. Therefore, the current study aims to determine the level of psychosocial functioning and delineate further the contribution of known and potential predictors among long-stay schizophrenia patients in a Malaysian mental institution. This research could provide evidence for future recommendations to integrate routine screening, early detection and intervention of psychosocial functioning for at-risk individuals, thereby contributing to a better prognosis for schizophrenia.

## Materials and Methods

### Study Design, Site and Population

A hospital-based, cross-sectional study was conducted among long-stay schizophrenia patients in the general psychiatry ward of the largest mental institution in Malaysia. This facility serves as a national tertiary psychiatric referral hospital for extended stays, further stabilisation and rehabilitation. Data were collected from February 2023 to May 2023.

### Sample Size and Inclusion and Exclusion Criteria

A meta-analytic approach was used to estimate the sample size for the current study. Across the dependent variables (employment status, severity of illness, depression, anxiety, and executive function), a review of the existing literature suggested that the weighted mean effect size was 0.3. With a power of 0.80 and considering a 10% dropout rate, this resulted in a required sample size of 95 participants.

The inclusion criteria were as follows:

- Patients who had schizophrenia formally diagnosed by a psychiatrist or medical officer based on the Diagnostic and Statistical Manual of Mental Disorders (32).
- Patients who had been occupying a psychiatric bed with a current duration of admission of six months or longer during the data collection period, which is considered a long-stay (6, 8).
- Patients aged 18 to 60 years. This age range was chosen because, in Malaysia, individuals below 18 years are classified as minors, while those aged 60 and above are considered an at-risk group who may have cognitive impairment due to other factors.

Meanwhile, the exclusion criteria were as follows:

- Patients suffering from a co-morbid psychiatric illness.
- Patients experiencing psychiatric diagnoses other than schizophrenia.
- Patients staying in the forensic psychiatric ward.
- Patients unable to provide informed consent.

### Study Procedure

Universal sampling was used for subject recruitment after screening all 38 general psychiatry wards in the study location for eligible participants. This sampling technique was adopted due to concerns that some participants might be psychotic and unable to provide informed consent, thus, limiting sample size fulfilment.

Medical records were examined, and face-to-face interviews with the respondents were conducted to obtain data regarding their socio-demographic factors and illness characteristics. Information such as the duration of stay and the types of antipsychotics prescribed were also retrieved from the medical records. By conducting the interviews and making observations, the researcher could also gauge the illness severity and psychopathology by filling out the Brief Psychiatric Rating Scale (BPRS). The participants also completed the Montreal Cognitive Assessment (MoCA) to evaluate their cognitive function. Lastly, the researcher filled out the Personal and Social Performance (PSP) scale with the assistance of staff members’ observations.

### Variables/Measurement Scales

The independent variables included socio-demographics (age, gender, ethnicity, education level, marital status, last employment status, and economic status), illness characteristics (psychopathology, number of previous psychiatric hospitalisations, age of onset, duration of illness, illness severity, type of antipsychotic prescribed, and duration of stay) and cognitive function. Meanwhile, the dependent variable was psychosocial functioning.

#### Psychopathology and Illness Severity (BPRS-18 Version)

The BPRS was developed to characterise psychopathology and assess its severity among individuals diagnosed with psychosis (33). It is clinician-rated and comprises 18 items assessing various psychiatric symptoms, rated on a severity scale from 1 (not present) to 7 (extremely severe). The overall score and the ratings on individual items are meaningful in capturing changes during treatment. While the original scale has inter-rater reliabilities ranging from 0.56 to 0.87, the locally validated Malay version also demonstrated fair internal consistency, with a Cronbach’s alpha coefficient of 0.75 (34). Fair concurrent validity was found when comparing it with the Positive and Negative Syndrome Scale (PANSS) and the Clinical Global Impression Scale (CGI) (35). Given these attributes and its ease of administration, the researcher chose BPRS to assess psychopathology and illness severity in the participants.

#### Cognitive Function (MoCA)

The MoCA is a brief 12-item cognitive screening tool that evaluates visuospatial and executive functioning, short-term memory, attention/concentration/working memory, language, and orientation. It operates on a 30-point system, with a cutoff score of 26 recommended to identify a normal cognitive state. Additionally, the severity of cognitive impairment can be categorised as mild (18 to 25), moderate (10 to 17), and severe (<10). The additional psychometric properties of MoCA include its sensitivity in testing executive function and its effectiveness in identifying mild cognitive impairment (36, 37). Its specificity for normal controls is 87%, with 90% sensitivity for detecting mild cognitive impairment, surpassing that of the Mini-Mental State Examination (36, 38). It is also reliable, with a Cronbach’s alpha value of 0.79 (39).

#### Psychosocial Functioning (PSP)

The PSP scale was developed to evaluate a patient’s psychosocial functioning across four subdomains: i) socially useful activities (including work and study); ii) personal and social relationships; iii) self-care and care for the personal environment; and iv) disturbing and aggressive behaviour (40). Each subdomain’s impairment is assigned a six-point rating, ranging from absent (0) to mild (1), manifest (2), marked (3), severe (4) and very severe (5). After scoring each of these four subdomains, raters are instructed to select a 10-point range within a 100-point scale, guided by the subdomain scores assigned during the assessment. All 10-point ranges on the scale are described in terms of two components: i) the scores assigned to the first three subdomains, and ii) the score assigned to the fourth subdomain. The first three areas contribute equally, while the fourth is given additional weight in determining the appropriate 10-point range. For the purpose of this study, the PSP total scores (ranging from 1 to 100) were derived from the sum of severity scores across the four subdomains, representing overall functioning. Higher PSP total scores indicate a higher level of functioning. The Cronbach’s alpha value for the PSP is 0.76 (40).

### Statistical Analysis

Data were analysed using the Statistical Package for the Social Sciences (SPSS) version 25 (IBM Corp., Armonk, NY, US). The distribution of all continuous variables was examined for normality via skewness, kurtosis, and histogram tests, all of which indicated normal distribution. Descriptive statistics were employed to describe the participants’ socio-demographic factors, illness characteristics, cognitive functioning, and psychosocial functioning.

The relationships between socio-demographics, illness characteristics, and cognitive functioning with psychosocial functioning (PSP score) were explored. The differences in PSP scores between categorical variables were tested using independent sample t-tests and one-way analysis of variance (ANOVA), while the correlation between the PSP scores and continuous variables was assessed using the Pearson correlation coefficient. Predictors of psychosocial functioning were determined using multiple linear regression analyses. Simple linear regression was first performed as a univariable exploration, and factors with a p-value of less than 0.05 were included in the variable selection process for the multivariable model using the stepwise method. Multicollinearity was assessed, and heteroskedasticity was checked using the Breusch–Pegan/Cook–Weisberg test. All tests were two-sided, and statistical significance was denoted by *p* < 0.05.

## Results

A total of 380 long-stay patients in 38 general psychiatry wards were identified and screened. Out of these, 111 patients were excluded due to co-morbid psychiatric conditions or a psychiatric diagnosis other than schizophrenia. This left a total of 269 long-stay schizophrenia patients (70.8%). Following further eligibility screening, 94 patients over the age of 60 and 22 patients who were unable to give informed consent due to irrelevant speech were excluded from the study. Another 15 patients had declined to participate. As a result, 138 long-stay schizophrenia patients were recruited for the study.

### Descriptive Analyses

Table 1 depicts the socio-demographic characteristics, illness characteristics, cognitive function and psychosocial functioning of the study participants. The mean age was 51.04 years [standard deviation (SD) = 7.17]. The majority were men (70.3%), Chinese (45.7%), had secondary education (73.2%), were single (72.5%), and had been employed either full-time, part-time or self-employed prior to admission (72.5%). Furthermore, 71.0% were under the B40 group (i.e., had a monthly household income of below RM4,850).

The mean age of illness onset was 27.51 years old (SD = 8.28), with a mean illness duration of 23.29 years (SD = 9.61). The mean duration of stay in the mental institution was 3,871 days (SD = 2536), or around 10 years. On average, patients had about seven previous psychiatric hospitalisations. Typical antipsychotics were the most commonly prescribed treatment (50.7%) in this population. Regarding illness severity, the mean BPRS total score was 27.12 (SD = 4.61). The respective BPRS mean scores for each psychopathology domain were as follows: positive symptoms [6.22 (SD = 2.62)]; mood disturbance [6.46 (SD = 1.74)]; negative symptoms [6.51 (SD = 1.91)]; activation [2.01 (SD = 0.09)]; resistance [2.40 (SD = 0.57)]; somatisation [2.60 (SD = 0.83)]; and orientation [1.00 (SD = 0.00)].

A total of 47.8% of the patients exhibited moderate cognitive impairments, while 16.7% had severe cognitive impairments. Lastly, the mean PSP score was 69.68 (SD = 15.48), translating to manifest, but not marked, difficulties in one or more domains (i.e., socially useful activities, personal and social relationships, self-care and care for the personal environment) or mild difficulties in the domain of disturbing and aggressive behaviour.

## Inferential Analyses

Table 2 depicts the relationship between socio-demographics, illness characteristics, cognitive function, and psychosocial functioning among long-stay schizophrenia patients. It was observed that females, those previously unemployed, and those with more severe cognitive impairments were significantly associated with poorer psychosocial functioning.

A weak negative correlation was found between psychosocial functioning and positive symptoms (coefficient: −0.266; *p* = 0.002), mood disturbance (coefficient: −0.242; *p* = 0.004), and resistance (coefficient: −0.240; *p* = 0.005). Conversely, a moderate negative correlation was observed between psychosocial functioning and age of onset (coefficient: −0.407; *p* < 0.001). Meanwhile, a strong negative correlation existed between psychosocial functioning and negative symptoms (coefficient: −0.726; *p* < 0.001). Moreover, a weak positive correlation was noted between psychosocial functioning and the duration of illness (coefficient: 0.379; *p* < 0.001).

Table 3 presents the predictors of psychosocial functioning among long-stay schizophrenia patients. The factors that were significant in the univariable exploration included gender (*p* = 0.004), educational levels (*p* = 0.028), last employment status (*p* = 0.008), positive symptoms (*p* = 0.002), mood disturbance (*p* = 0.004), negative symptoms (*p* < 0.001), resistance (*p* = 0.005), illness severity (*p* < 0.001) and levels of cognitive function (*p* < 0.001). In the final multivariate analyses, the significant predictors for psychosocial functioning among long-stay schizophrenia patients, after controlling for the potential confounders, included only gender (*p* < 0.001), last employment status (*p* = 0.047), negative symptoms (*p* < 0.001), duration of illness (*p* = 0.004), and cognitive function (*p* < 0.001).

Females were found to have lower PSP total scores by 7.32 points compared to males (*p* < 0.001). Those previously unemployed had PSP total scores that were 3.67 points lower than those who were previously employed (*p* = 0.047). For illness characteristics, a unit increase in negative symptoms was associated with a decrease of 4.18 points in PSP total scores (*p* < 0.001). Furthermore, a unit longer in the duration of illness would reduce the PSP total scores by 0.60 points (*p* = 0.004). Participants with severe cognitive impairment had PSP total scores that were 9.80 points lower than those with mild cognitive function (*p* < 0.001). The *R*^2^ of the final multivariable model was 0.6576, indicating that the model explained 65.76% of the variance in psychosocial functioning.

## Discussion

This study determined the level of psychosocial functioning and examined the contribution of known and potential predictors in a sample of long-stay schizophrenia patients from a Malaysian mental institution. First, the results corroborate prior research findings that long-term hospitalisation leads to pronounced cognitive and psychosocial impairments. Patients with schizophrenia may require hospitalisation during acute episodes or crises. Despite a remarkable reduction in the duration of hospital stays due to improved care from community psychiatric services, many patients continue to experience prolonged stays. While a study concluded there was minimal evidence linking long or short hospitalisation to cognitive and functional changes—attributing declines to the reasons for hospitalisations (30)—prolonged hospitalisation is arguably regarded as counterproductive and is one of the associated factors contributing to disability in schizophrenia (19).

In this study’s sample, the measured psychosocial functioning was poorer (mean PSP total score of 69.68) compared to study subjects from community-dwelling schizophrenia patients (mean PSP total score of 84.37) (41). These difficulties reflect challenges in performing daily activities, social interactions and employment, highlighting the impacts of prolonged institutionalisation on psychosocial outcomes. The influence of care settings on cognitive function progress remains controversial; community-dwelling patients certainly have better opportunities for cognitive remediation, while long-stay patients typically exhibit poorer functioning (42). More than half of the participants experienced moderate to severe cognitive deficits. None of them had normal cognitive function despite being under 60 years of age, suggesting that prolonged stay in the hospital has not only failed to improve this aspect of their illness but may have worsened it, as suggested by previous studies (43, 44).

Therefore, it is crucial to determine predictors of psychosocial functioning for targeted prevention and early intervention. Despite many studies exploring this area, only a few have focused on long-stay schizophrenia patients, and none have investigated this specifically among Asians. The current study builds on earlier findings regarding the clinical importance of several psychosocial functioning predictors in what is believed a distinct subset of schizophrenia patients. These include identifying gender, employment status, negative symptoms, duration of illness, and cognitive function.

Many researchers agree that gender differences play a role in the clinical and functional outcomes of schizophrenia (45). In contrast to well-documented findings indicating that schizophrenia outcomes are poorer in male patients, who tend to have an earlier age of onset, worse premorbid adjustment, and a more severe disease course, this study’s female patients had lower psychosocial function scores (46, 47). This may be attributed to the higher number of elderly females and those previously unemployed in our sample population. Decreased oestrogen levels after menopause can impair cognition, especially in memory and executive function domains, which subsequently influence psychosocial functioning (48). While social and intellectual premorbid adjustments predict cognitive and functional outcomes in schizophrenia, postmorbid negative symptoms appear to play a mediating role (49, 50).

Generally, people with schizophrenia experience long-lasting negative symptoms throughout their illness. Moreover, a longer duration of illness is associated with more prominent negative symptoms and has been found to predict lower psychosocial functioning in this study’s population (51). This aligns with observations that long-term institutionalised schizophrenia patients, especially those who are geriatric and chronically ill, exhibit more significant negative symptoms than younger and community-dwelling patients (52). Researchers have characterised a difference in the negative symptom profiles of people with schizophrenia who are in long-term hospital-based care compared to those living in the community, with severe asociality being the most characteristic symptom of prolonged hospitalisation (53). A previous study investigating the additional influence of negative symptoms on real-world functioning in people with schizophrenia concluded that diminished expression and avolition-apathy contribute substantial additional variance in predicting both real-world functioning and employment outcomes after accounting for neurocognition and functional capacity (54). Given these insights, the management of negative symptoms in long-stay schizophrenia patients, especially those with a protracted history of illness, necessitates comprehensive strategies, including medication adjustments, psychosocial interventions, family support, and cognitive remediation, to improve patients’ functional outcomes (24, 55–57).

To date, neurocognitive and social cognitive impairments have been identified as determinants of disability in schizophrenia, including vocational drift, emphasising the importance of cognitive assessment and remediation (23, 58). Meanwhile, premorbid employment status may indicate levels of social adjustment and intellectual function before disease onset, both of which influence psychosocial functioning among schizophrenia patients (49). In the present study, being unemployed prior to the current extended hospital stay correlated with lower psychosocial functioning, further confirming the need to pay attention to this group of patients with poor premorbid functions. There is a pressing need to strengthen vocational rehabilitation in the local setting to equip patients with job skills and opportunities, fostering a sense of purpose and social integration (15, 17, 41, 59). By empowering patients through employment and vocational support, mental health professionals can also enhance their psychosocial functioning and overall well-being (15, 17, 41, 59).

Community-based care offers better opportunities for social integration and professional competence, potentially leading to improved outcomes. The findings emphasise the necessity for comprehensive interventions targeting long-stay schizophrenia patients. Treatment strategies should incorporate cognitive remediation, psychosocial rehabilitation and symptom management to enhance functional outcomes. Community-based care and support programmes are crucial in preventing prolonged hospitalisation and promoting better long-term outcomes for individuals with schizophrenia. In adopting a holistic approach to the treatment and management of psychosocial functioning of long-stay schizophrenia patients, the following strategies should be considered: individualised treatment plans tailored to the unique needs and challenges of each patient, accounting for factors such as gender, cognitive abilities and negative symptoms. Vocational rehabilitation programmes (60–62), social skills training (63) and cognitive remediation (64, 65) should be integrated into treatment plans to improve functional outcomes, particularly for those who are unemployed and facing cognitive impairment. Cognitive remediation has also been shown to effectively treat negative symptoms (66, 67). Regular psychosocial interventions, family support and community integration efforts are essential to address social withdrawals and deficits. Early intervention and continuous monitoring can help prevent symptom worsening and cognitive decline over time.

This current study is the first research conducted in a local setting exploring predictors of psychosocial functioning among long-stay schizophrenia patients in the country’s largest mental institution, with a relatively significant number of study subjects. Nevertheless, this study has some limitations that need to be addressed. First, it is a cross-sectional design that does not allow for inferring causal links. Second, the final multivariable model explained 65.76% of the variance in psychosocial functioning. While this indicates that the included predictors account for a substantial portion of the variance, other factors influencing psychosocial functioning were not explored in this study. Future research could investigate additional variables, such as social support, family dynamics, insight, coping mechanisms, as well as perceived and self-stigma, to provide a more comprehensive understanding of the predictors of psychosocial functioning in long-stay schizophrenia patients (68–70). The current study used a universal sampling method to achieve an adequate sample size, meaning that the participants were not randomly selected. Furthermore, the study included only one mental institution in Malaysia, which might limit the generalisability of the findings. Hence, future research should employ longitudinal designs, consider a broader range of variables and adopt larger samples in multicentre settings. Lastly, including community-dwelling schizophrenia patients as a comparison would yield a more comprehensive understanding of the unique challenges faced by long-stay patients.

## Conclusion

This study highlights that long-stay schizophrenia patients in mental institutions experience substantial difficulties in psychosocial functioning. By identifying gender, previous employment status, negative symptoms, illness duration and cognitive function as predictors of psychosocial functioning, targeted interventions can be developed to improve functional outcomes among at-risk populations. Future research should continue to explore effective strategies to support this distinct patient population and promote their overall well-being within mental institutions and beyond.

## Figures and Tables

**Table 1:** Socio-demographics, illness characteristics and cognitive and psychosocial functions of long-stay schizophrenia patients (n = 138)

Independent variable	Mean (SD)	n (%)
Age (years)	51.04 (7. 17)	

Gender		

Male		97 (70.3)

Female		41 (29.7)

Ethnicity		
Malay		58 (42. 1)
Chinese		63 (45.7)
Indian		15 (10.9)
Others		2 (1.4)

Education level		
None		0
Primary education		34 (24.6)
Secondary education		101 (73.2)
Tertiary education		3 (2.2)

Marital status		
Single		100 (72.5)
Married or living in union		26 (18.8)
Separated or widowed or divorced		12 (8.7)

Last employment status		
Employed (full-time or part-time or self-employed)		100 (72.5)
Unemployed		38 (27.5)

Monthly household income		
T20: exceeds RM10,960 per month		6 (4.3)
M40: between RM4,851 to RM10,960 per month		34 (24.6)
B40: below RM4,850 per month		98 (71.0)

Psychopathology severity based on BPRS		
Positive symptoms	6.22 (2.62)	
Mood disturbance	6.46 (1.74)	
Negative symptoms	6.51 (1.91)	
Activation	2.01 (0.09)	
Resistance	2.40 (0.57)	
Somatisation	2.60 (0.83)	
Orientation	1.00 (0.00)	

Number of previous psychiatric hospitalisations	6.57 (6.60)	

Age of schizophrenia onset (years)	27.51 (8.28)	

Duration of illness (years)	23.29 (9.61)	

Illness severity (BPRS total score)	27. 12 (4.61)	

Type of antipsychotic prescribed		
Atypical		6 (4.3)
Typical		70 (50.7)
Combination (atypical + typical)		62 (44.9)

Duration of stay (days)	3871.00 (2536.00)	

Cognitive function (MoCA total scores)	14.93 (5.43)	

Level of cognitive function		
Normal		0
Mild cognitive impairment		49 (35.5)
Moderate cognitive impairment		66 (47.8)
Severe cognitive impairment		23 (16.7)

Psychosocial functioning (PSP total scores)	69.68 (15.48)	

**Table 2:** Relationships between socio-demographics, illness characteristics and cognitive function with psychosocial functioning

Variable		Psychosocial functioning	

		PSP total scores, mean (SD)	*p*-value
Gender	Male	72. 18 (14.87)	0.004^b^
	Female	63.78 (16.68)	

Ethnicity	Malay	68.81 (16.16)	0.517^c^
	Chinese	69.37 (15.86)	
	Indian	72.47 (15.39)	
	Others	84.00 (2.83)	

Educational level	Primary	65.82 (15.74)	0.056^c^
	Secondary	70.48 (15.73)	
	Tertiary	86.67 (3.22)	

Marital status	Single	70.27 (15.37)	0.362^c^
	Married	65.96 (17.53)	
	Separated/widowed	72.83 (15.97)	

Last employment status	Employed	71.86 (14.08)	0.008^b^
	Unemployed	63.95 (18.76)	

Economic status	T20	81.17 (6.97)	0.128^c^
	M40	71.26 (13.29)	
	B40	68.43 (16.77)	

Type of antipsychotic prescribed	Typical	63.00 (14.30)	0.510^c^
	Atypical	69.34 (16.60)	
	Combination	70.71 (15.16)	

Cognitive impairment category	Mild	79.59 (11.46)	<0.001^c^
	Moderate	69.39 (13.25)	
	Severe	49.39 (10.39)	

**Variable**		**Psychosocial functioning**	

		**Pearson coefficient**	***p*** **-value**

Age		0.013	0.878^a^
Type of psychopathologies:			
Positive symptoms		−0.266	0.002^a^
Mood disturbance		−0.242	0.004^a^
Negative symptoms		−0.726	<0.001^a^
Activation		−0.036	0.674^a^
Resistance		−0.240	0.005^a^
Somatisation		−0.085	0.319^a^
Age of onset		−0.407	<0.001^a^
Duration of illness		0.379	<0.001^a^
Illness severity		0. 111	0.193^a^
Duration of stay		−0.179	0.047^a^

Notes:

a Pearson correlation;

b Independent sample *t*-test;

c One-way ANOVA

**Table 3:** Predictors of psychosocial functioning among long-stay schizophrenia patients

Variable	Standardised beta coefficient	Simple linear regression		Multiple linear regression	

		Coef (95% CI)	*p*-value	Adj coef (95% CI)	*p*-value
Age	−0.007	0.03 (−0.35, 0.40)	0.878		

Gender	−0.210				
Male		Ref		Ref	
Female		−8.39 (−14.08, −2.71)	0.004	−7.32 (− 10.82, −3.81)	<0.001

Ethnicity	−0.023				
Malay		Ref			
Chinese		0.55 (−5.16, 6.27)	0.848		
Indian		3.66 (−5.44, 12.76)	0.428		
Others		15. 19 (−7.40, 37.78)	0.186		

Educational level	0.031				
Primary		Ref	0.136		
Secondary		4.65 (−1.47, 10.78)			
Tertiary		20.84 (2.23, 39.45)	0.028		

Marital status	0.007				
Single		Ref			
Married		−4.31 (−11.20, 2.59)	0.219		
Separated/Widowed/Divorced		2.56 (−7.01, 12. 13)	0.597		

Last employment status	−0.113				
Employed		Ref	0.008	Ref	0.047
Unemployed		−7.91 (− 13.75, −2.07)		−3.67 (−7.29, −0.05)	

Economic status	0.035				
T20		Ref			
M40		−9.90 (−23.67, 3.86)	0.157		
B40		−12.74 (−25.81, 0.33)	0.056		

Types of psychopathology					
Positive symptoms	0.021	−1.61 (−2.60, −0.62)	0.002	−4. 18 (−5.23, −3. 13)	<0.001
Mood disturbance	−0.081	−2.20 (−3.69, −0.70)	0.004		
Negative symptoms	−0.511	−6.02 (−6.98, −5.05)	<0.001		
Activation	0.021	−6.73 (−38.27, 24.81)	0.674		
Resistance	−0.056	−6.62 (−11.17, −2.07)	0.005		
Somatisation	−0.080	−1.63 (−4.84, 1.59)	0.319		

Number of hospitalisations	0.041	0.22 (−0.19, 0.63)	0.286		

Age of onset	0.113	0.21 (−0. 11, 0.54)	0.194		

Duration of illness	0.163	−0.16 (−0.44, 0. 12)	0.264	−0.60 (− 1.00, −0. 19)	0.004

Illness severity	−0.064	−1.91 (−2.39, −1.42)	<0.001		

Types of antipsychotic prescribed	−0.022				
Typical		Ref			
Atypical		6.34 (−7.02, 19.70)	0.349		
Combination		7.71 (−5.72, 21. 14)	0.258		

Duration of stay	−0.007	−0.01 (−0.01, 0.00)	0. 144		

Cognitive function category	−0. 112				
Mild		Ref		Ref	
Moderate		−10.20 (−14.75, −5.65)	<0.001	−9.80 (−14.69, −4.92)	<0.001
Severe		−30.20 (−36.30, −24. 10)	<0.001		

Notes: Coef = beta coefficient; Adj Coef = adjusted beta coefficient; CI = confidence interval; Ref = reference. Multicollinearity was not found, and assumptions of heteroskedasticity were fulfilled. Adjusted *R^2^ =* 0.6576
